# Comprehensive evaluation of the implementation of episignatures for diagnosis of neurodevelopmental disorders (NDDs)

**DOI:** 10.1007/s00439-023-02609-2

**Published:** 2023-10-27

**Authors:** Edoardo Giuili, Robin Grolaux, Catarina Z. N. M. Macedo, Laurence Desmyter, Bruno Pichon, Sebastian Neuens, Catheline Vilain, Catharina Olsen, Sonia Van Dooren, Guillaume Smits, Matthieu Defrance

**Affiliations:** 1 Interuniversity Institute of Bioinformatics in Brussels, Université Libre de Bruxelles-Vrije Universiteit Brussel, Brussels, Belgium; 2 Center for Human Genetics, Hôpital Erasme, Hôpital Universitaire de Bruxelles, Université Libre de Bruxelles, Brussels, Belgium; 3 Department of Genetics, Hôpital Universitaire Des Enfants Reine Fabiola, Hôpital Universitaire de Bruxelles, Université Libre de Bruxelles, Brussels, Belgium; 4 Clinical Sciences, Research Group Reproduction and Genetics, Brussels Interuniversity Genomics High Throughput Core (BRIGHTcore), Vrije Universiteit Brussel (VUB), Universitair Ziekenhuis Brussel (UZ Brussel), Brussels, Belgium; 5 Clinical Sciences, Research Group Reproduction and Genetics, Centre for Medical Genetics, Vrije Universiteit Brussel (VUB), Universitair Ziekenhuis Brussel (UZ Brussel), Brussels, Belgium

## Abstract

Episignatures are popular tools for the diagnosis of rare neurodevelopmental disorders. They are commonly based on a set of differentially methylated CpGs used in combination with a support vector machine model. DNA methylation (DNAm) data often include missing values due to changes in data generation technology and batch effects. While many normalization methods exist for DNAm data, their impact on episignature performance have never been assessed. In addition, technologies to quantify DNAm evolve quickly and this may lead to poor transposition of existing episignatures generated on deprecated array versions to new ones. Indeed, probe removal between array versions, technologies or during preprocessing leads to missing values. Thus, the effect of missing data on episignature performance must also be carefully evaluated and addressed through imputation or an innovative approach to episignatures design. In this paper, we used data from patients suffering from Kabuki and Sotos syndrome to evaluate the influence of normalization methods, classification models and missing data on the prediction performances of two existing episignatures. We compare how six popular normalization methods for methylarray data affect episignature classification performances in Kabuki and Sotos syndromes and provide best practice suggestions when building new episignatures. In this setting, we show that Illumina, Noob or Funnorm normalization methods achieved higher classification performances on the testing sets compared to Quantile, Raw and Swan normalization methods. We further show that penalized logistic regression and support vector machines perform best in the classification of Kabuki and Sotos syndrome patients. Then, we describe a new paradigm to build episignatures based on the detection of differentially methylated regions (DMRs) and evaluate their performance compared to classical differentially methylated cytosines (DMCs)-based episignatures in the presence of missing data. We show that the performance of classical DMC-based episignatures suffers from the presence of missing data more than the DMR-based approach. We present a comprehensive evaluation of how the normalization of DNA methylation data affects episignature performance, using three popular classification models. We further evaluate how missing data affect those models’ predictions. Finally, we propose a novel methodology to develop episignatures based on differentially methylated regions identification and show how this method slightly outperforms classical episignatures in the presence of missing data.

**Supplementary Information:**

The online version contains supplementary material available at 10.1007/s00439-023-02609-2.

## Background

DNA methylation (DNAm) of the 5th carbon of cytosine sitting in cytosine-guanine (CpG) context is the most studied epigenetic process. Identification of differentially methylated cytosines (DMCs) is a promising area to improve diagnosis and gain knowledge on the etiology of diseases (Reichard and Zimmer-Bensch 2021; Mc Auley 2021). Unique methylation patterns allowing to discriminate between cases and control are termed episignatures (Malouf et al. 2016; Grafodatskaya et al. 2013). They are often deemed to be secondary epivariations (i.e., caused by sequence variants) as opposed to primary epivariations (i.e., caused by stochastic errors of the methylation machinery) (Horsthemke 2006). In the context of rare neurodevelopmental disorders (NDDs), episignatures in combination with classification models have emerged as a powerful tool to enable patients’ diagnosis and evaluate the pathogenicity of variants of unknown significance (VUS) (Choufani et al. 2015; Levy et al. 2022). To this date, more than 60 episignatures associated with neurodevelopmental disorders have been published. The majority of those have been developed from methylation data generated with the Illumina Infinium Methylation BeadChips 450 K and EPIC. In essence, episignature discovery derives from epigenome-wide association study (EWAS). Identification of DMCs can be done through group comparison by means of a statistical method. Classically, linear regression or Mann–Whitney *U* test and the addition of an effect threshold on the difference between controls and cases beta-values are applied to highlight DMCs. The resulting set of DMCs beta-values is then used to train a classification model such as support vector machines (SVM), random forest (RF) or penalized logistic regression (PLR) (Grafodatskaya et al. 2013; Choufani et al. 2015; Butcher et al. 2017). Despite their increasing popularity, few guidelines have been emitted on how to generate episignatures with the exception of an article by Chater-Diehl and colleagues that discusses the influence of the size of the training data, its origin, and effect size (Chater-Diehl et al. 2021). The absence of a gold standard methodology has led to the generation of different episignatures for similar pathologies (Choufani et al. 2015; Butcher et al. 2017; Aref-Eshghi et al. 2020), and raised the question of reproducibility and adaptability to new datasets as several key parameters affect the generation of DNAm data through Infinium BeadChips. Indeed, due to array design, it was shown that normalization plays a crucial role in data preprocessing (Dedeurwaerder et al. 2011; Bizet et al. 2022). One of the main tools to preprocess Infinium BeadChips data is the *minfi* package in *R* (Fortin et al. 2017; Aryee et al. 2014). This package includes different normalization methods for methylation data that are considered the gold standards: Beta MIxture Quantile (BMIQ) dilation normalization (Touleimat and Tost 2012), Functional normalization (Funnorm) (Fortin et al. 2014), Noob (normal-exponential out-of-band) (Triche et al. 2013), Subset-quantile Within Array Normalization (SWAN) (Maksimovic et al. 2012), as well as the reimplementation of the Illumina GenomeStudio normalization method, and a raw normalization (no normalization). Therefore, there is a need to evaluate how those different normalization methods affect episignature discovery and robustness. In addition, since the vast usage of Infinium HumanMethylation 450 K BeadChip (IM450K), there have been two iterations of the Illumina microarray: Infinium HumanMethylation EPIC BeadChip (IMEPIC) and Infinium HumanMethylation EPICv2 BeadChip (IMEPICv2). Despite high inter-array robustness and reproducibility across tissues (Cheung et al. 2020; Full article: Comparison of Illumina 450K and EPIC arrays in placental DNA methylation. xxxx; Moran et al. 2016), each new version of the arrays involves CpGs addition and removal. Therefore, CpGs from an episignature produced on one technology are not always present on another version. This issue also arises due to batch-effect related probes removal in the preprocessing steps. Those missing values require the use of imputation for the classifier to work which can lead to noise and error. An alternative strategy to overcome those limitations would be considering methylation levels in regions of consecutive DMCs called differentially methylated regions (DMRs). Indeed, DMRs have been identified in several neurodevelopmental and congenital disorders (Barbosa et al. 2018; Kagami et al. 2021) and showed diagnostic utility even in a single patient setting (Grolaux et al. 2022). Thus, using DMRs median methylation levels would mitigate the presence of missing values and is more likely to stay compatible with new microarrays iterations. Finally, three main classification models (SVM, RF, and PLR) are usually adopted to evaluate the capacity of an episignature to differentiate between cases and controls (Hannon et al. 2021). However, their performances have never been thoroughly assessed. In this work, we perform the first comprehensive evaluation of how normalization methods and classification algorithms affect prediction performance, based on two episignatures for Kabuki and Sotos syndromes (Choufani et al. 2015; Butcher et al. 2017). We further show that missing data affect classical DMC-based episignature performances and propose two new episignatures based on the detection of DMRs instead of DMCs to mitigate this effect. In conclusion, we provide researchers with guidelines on the preprocessing steps to consider when implementing new episignatures as well as a new paradigm to build DMR-episignatures (DMR-esigs) instead of the classically used DMC-based episignatures (DMC-esigs).

## Results

### Testing Kabuki and Sotos DMCs episignatures

The DMC-esig for Kabuki (Kabuki^DMC^) and Sotos (Sotos^DMC^) have been described before in two separate studies (Choufani et al. 2015; Butcher et al. 2017). They are composed of, respectively, 113 and 7,085 CpGs identified using Mann–Whitney *U* test and a threshold on effect size. *Idat* files from the original studies were normalized using 6 different methods available in *minfi*: Swan, Funnorm, Raw, Illumina, Quantile, and Noob. We showed that unsupervised hierarchical clustering was able to cluster cases and controls of the training sets for almost every normalization method using both signatures (Additional Figs. 1 and 2). Indeed, we noticed that both Sotos^DMC_Raw^ and Sotos^DMC_Swan^ failed to completely separate patients and controls, as two cases (GSM1920409, GSM1920415) were clustered with the controls. Moreover, those two cases are clustered together even when normalizing data with the other methods and noticeably display a different methylation profile than the rest of the case-cohort (Additional Fig. 2). Interestingly, those two samples are described in the original paper as sharing the same mutation at the end of exon 22 of NSD1, which could explain this behavior. Dimensionality reduction using UMAP allowed clear separation of the different samples for both signatures (Additional Figs. 1 and 2).

### Building Kabuki and Sotos DMRs

**Fig. 1:**
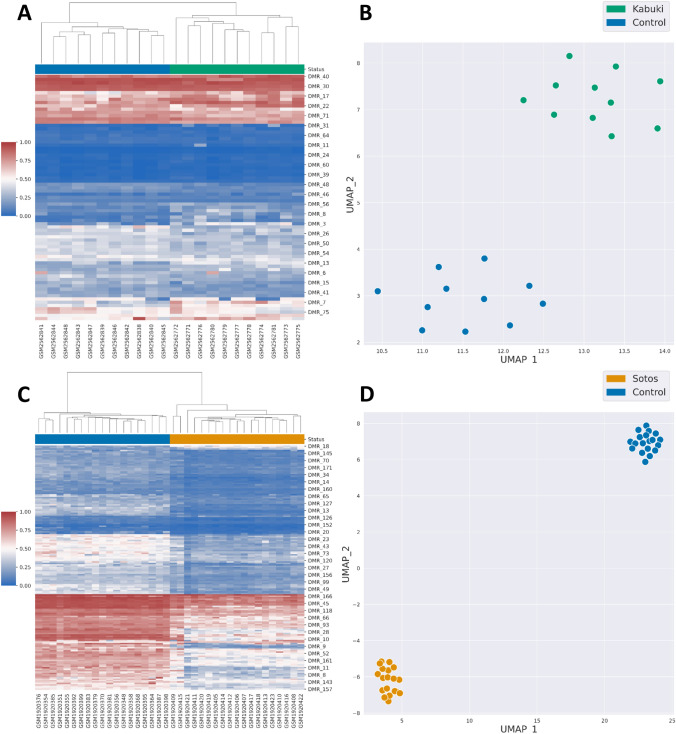
In **A** and **C**, the heatmaps and hierarchical clustering of 22 and 38 samples of, respectively, Kabuki (cases: green, controls: blue) and Sotos (cases: orange, controls: blue) training sets show a perfect cluster of cases and controls in two different clusters. In the heatmaps, the red indicates a high methylation level, while the blue indicates a low methylation level. The clustering was performed using Sotos^DMR_Illumina^ and Kabuki^DMR_Illumina^. The same DMR-esigs were used to plot Kabuki and Sotos samples in a lower, two-dimensional space using the UMAP dimensionality reduction method (**B** and **D**, respectively). The samples clearly define two different groups after UMAP in both syndromes

To build DMR-esig for Sotos (Sotos^DMR^) and Kabuki (KabukiDMR) syndromes, we applied the bumphunter method from champ. DMR on the training data from the two aforementioned studies normalized with the 6 different methods in minfi. For each DMR found, according to each normalized dataset, several parameters are reported in Additional Files 1 and 2 for Sotos and Kabuki syndrome, respectively, including the length of the DMR (width) and the number of CpGs included in the region (L). The resulting number of DMRs varied between both syndromes and normalization methods (Additional Fig. 3). The mean number of DMRs identified was higher in Sotos (*n* = 51) than in Kabuki (*n* = 38). However, this was mainly due to the Illumina normalized dataset that showed the highest number of DMRs in both syndromes (Kabuki = 75, Sotos = 171, Additional Fig. 3). For each dataset, the median methylation level per DMR was computed using the beta-values from the CpGs located within the region. The resulting set of median DMR methylation values constitutes the DMR-esig. We applied unsupervised hierarchical clustering and showed that similarly to DMC-esig, Sotos^DMR^ and KabukiDMR were able to discriminate between cases and controls using all the normalization methods (Fig. 1 and Additional Fig. 4). Notably, median methylation values of DMRs in Sotos cases were lower than in controls. UMAP dimensionality reduction techniques allowed a clear separation between all samples in the training sets (Fig. 1, Additional Figs. 4 and 5). Similarly to what is described in the section above, the two cases (GSM1920409, GSM1920415) in the Sotos cohort were still clustered together in the heatmap using Sotos^DMR^, demonstrating that the DMR-esig is still able to capture the different methylation profiles of these two samples (Fig. 1). Furthermore, Kabuki samples in the UMAP generated with DMR-esigs are less separated than in the UMAP generated with DMC-esigs. This can be due to the presence of different information captured by the DMRs than the DMCs and can result in a different arrangement of points in the reduced-dimensional space.

## Classification results and prediction probabilities

**Fig. 2:**
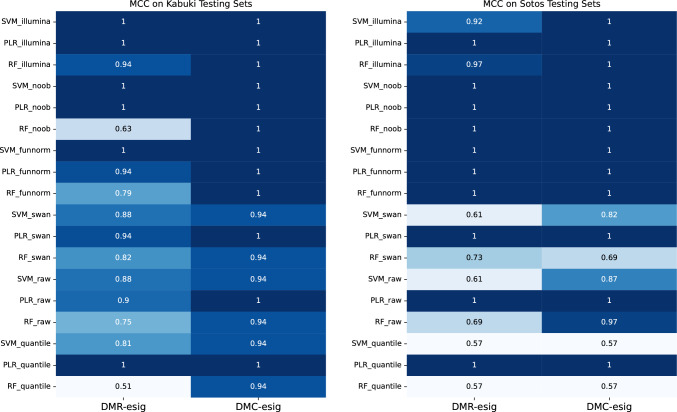
The heatmaps represent the MCC values on Kabuki (left) and Sotos (right) Testing Sets composed of both public IM450K samples and IMEPIC samples generated at Erasme Hospital. In the heatmaps, the values represent the MCC computed after the predictions given by the three models tested in this study (SVM, PLR, RF) when trained on DMR-esig and DMC-esig generated on training sets previously normalized with the six different normalization methods available in minfi library. The models were tested on the testing sets normalized with the same normalization method adopted during the training procedure. According to the performances in the heatmaps, we saw that the best models for DMR-esig were PLR and SVM for Sotos and Kabuki syndromes, respectively. With the DMC-esig, PLR was the best model for Sotos and Kabuki syndromes. Similarly, this also highlights the three best normalization methods (Illumina, Noob and Funnorm) in all configurations

Results from the previous section led to the training of 18 classifiers per syndrome. We trained random forest (RF), support vector machine (SVM), and penalized logistic regression (PLR) models on all six normalized training sets, using the DMC-esig and the corresponding DMR-esig (see Methods). After performing LOOCV to find the optimal hyperparameters, the trained SVM, RF, and PLR classifiers were tested on both IM450K testing set from the original studies (Choufani et al. 2015; Butcher et al. 2017) and IMEPIC testing set from the ULB Erasme Hospital Center for Human Genetics. Performances of the classifiers were evaluated using MCC. In the case of Sotos syndrome, all models achieved a MCC of 1 when trained on the DMC-esig. However, only PLR achieved a MCC of 1 regardless of the normalization method used, showing higher robustness to normalization methods than SVM and RF. Indeed, SVM and RF achieved maximum performance only with Illumina, Noob, and Funnorm normalization methods (Fig. 2). Our models trained on DMR-esig for Sotos syndrome shared similar behavior. PLR was again the best model, showing high robustness to the normalization method and achieving an MCC of 1 for all normalization methods. SVM and RF were able to reach an MCC of 1 only with Noob and Funnorm normalization methods (Fig. 2). Concerning Kabuki syndrome, among the models trained on DMC-esig, PLR was able to reach an MCC of 1 in both testing sets for all the normalization methods, again showing overall robustness to normalization methods. SVM and RF reached an MCC of 1 when using Funnorm, Illumina, and Noob functions. Concerning our models trained on DMR-esig, when tested on both testing sets, SVM reached an MCC of 1 when using Funnorm, Illumina and Noob normalization methods. PLR was able to reach an MCC of 1 only with Noob and Illumina functions, while RF never reached an MCC of 1 (Fig. 2).

Thus, per syndrome, we extracted the best-performing model and the three best normalization methods for both DMC-esig and DMR-esig. For Sotos syndrome, PLR was the best-performing model for both DMR-esig and DMC-esig. For Kabuki syndrome, PLR and SVM were the best-performing models for DMC-esig and DMR-esig, respectively. Illumina, Noob, and Funnorm were chosen as the best normalization methods in all configurations according to the MCC computed (Fig. 2). We then compared the performances of those 6 models using testing datasets with an increasing number of missing CpGs (0, 5, 10, 20, and 30%). We showed that the DMR-based models started to outperform the DMC models at 30% of missing CpGs for both Kabuki and Sotos datasets. The higher robustness of Sotos DMC-esig is likely due to the fact that the Sotos DMC-esig is composed of a large number of probes (> 7000) and the size effect between case and controls for Sotos disease is usually much higher (> 30%) than for Kabuki (Table 1).

**Table 1:** A represents the mean MCC (± Std) on Kabuki Testing Sets, while B represents the mean MCC (± Std) on Sotos testing sets at different levels of missing CpGs (0 to 30%), according to the best models and normalization methods previously selected (Fig. 2). The scenario of 0% of probes removal refers to the imputation of missing probes due to low-quality signal or new array version. This highlights the higher robustness of the DMR models to missing data compared to the DMC models

	Missing CpGs				
	0%	5%	10%	20%	30%
A—Kabuki					
DMR^SVM_Funnorm^	1.000 (± 0.000)	0.988 (± 0.025)	0.994 (± 0.019)	0.962 (± 0.061)	0.975 (± 0.052)
DMR^SVM_Illumina^	1.000 (± 0.000)	1.000 (± 0.000)	0.994 (± 0.019)	0.977 (± 0.030)	0.984 (± 0.036)
DMR^SVM_Noob^	1.000 (± 0.000)	1.000 (± 0.000)	0.994 (± 0.018)	0.973 (± 0.087)	0.954 (± 0.074)
DMC^PLR_Funnorm^	1.000 (± 0.000)	1.000 (± 0.000)	1.000 (± 0.000)	1.000 (± 0.000)	0.923 (± 0.190)
DMC^PLR_Illumina^	1.000 (± 0.000)	1.000 (± 0.000)	1.000 (± 0.000)	1.000 (± 0.000)	0.961 (± 0.068)
DMC^PLR_Noob^	1.000 (± 0.000)	1.000 (± 0.000)	1.000 (± 0.000)	1.000 (± 0.000)	0.978 (± 0.038)
B—Sotos					
DMR^PLR_Funnorm^	1.000 (± 0.000)	1.000 (± 0.000)	0.997 (± 0.008)	1.000 (± 0.000)	1.000 (± 0.000)
DMR^PLR_Illumina^	1.000 (± 0.000)	1.000 (± 0.000)	1.000 (± 0.000)	1.000 (± 0.000)	1.000 (± 0.000)
DMR^PLR_Noob^	1.000 (± 0.000)	1.000 (± 0.000)	1.000 (± 0.000)	1.000 (± 0.000)	1.000 (± 0.000)
DMC^PLR_Funnorm^	1.000 (± 0.000)	1.000 (± 0.000)	1.000 (± 0.000)	1.000 (± 0.000)	0.976 (± 0.028)
DMC^PLR_Illumina^	1.000 (± 0.000)	1.000 (± 0.000)	1.000 (± 0.000)	1.000 (± 0.000)	0.985 (± 0.022)
DMC^PLR_Noob^	1.000 (± 0.000)	1.000 (± 0.000)	1.000 (± 0.000)	1.000 (± 0.000)	0.986 (± 0.021)

### Specificity of Sotos^DMR^ and Kabuki^DMR^ episignatures

**Fig. 3:**
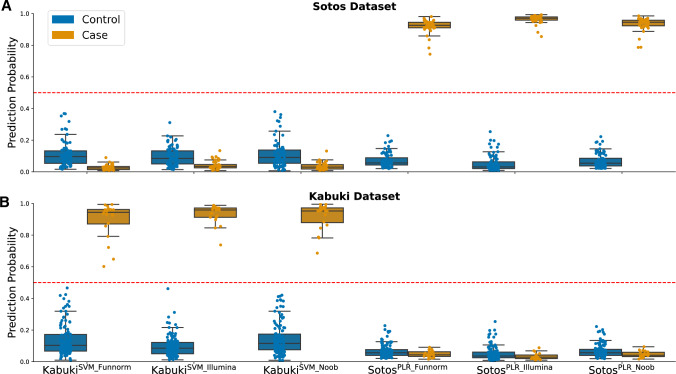
The entire Kabuki and Sotos datasets used during the training and testing procedures adopted to build the corresponding classifiers are used to assess if the classifiers are specific. Each point in the plots above corresponds to one sample (either control or case) belonging to the Kabuki (**B**) or Sotos (**A**) dataset. The best DMR-esig Kabuki and Sotos predictive models (SVM and PLR, respectively) were used to classify each sample present in both Kabuki and Sotos datasets normalized with the three best normalization methods (Illumina, Noob, or Funnorm). On the y-axis, the prediction probability assigned to each sample in all the settings is shown. The models are 100% specific and do not make overlapping predictions using the default threshold of 0.5: all the samples present in the Sotos dataset are classified as controls by the three Kabuki classifiers, and vice versa

In order to verify the specificity of our models trained on DMR-esig for Sotos and Kabuki, we tested the best Sotos predictive model (PLR) on the Kabuki cohort (training set + testing sets) and the best Kabuki predictive model (SVM) on the Sotos cohort. All the samples in the Kabuki dataset were predicted as controls by the best Sotos predictive model and all the samples in the Sotos dataset were predicted as controls by the best Kabuki predictive model, regardless of the normalization method adopted, thus showing high inter-syndrome specificity (Fig. 3).

**Fig. 4:**
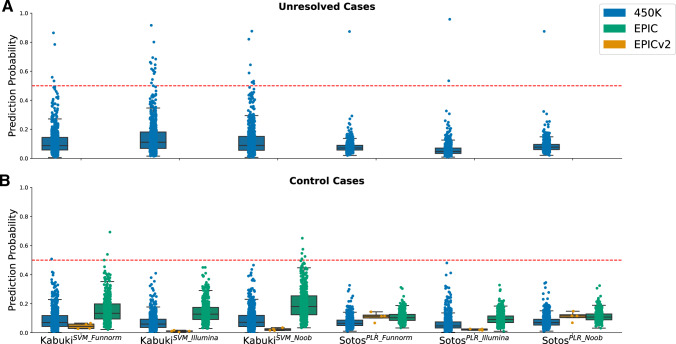
**A** The same six models adopted in Fig. 3 have been used to assess the specificity of the Sotos and Kabuki best predictive models have been applied to a cohort of 489 IM450K unresolved cases (GSE89353). These samples were normalized with the BMIQ method. **B** The same six models have been applied on a larger cohort of control samples composed of 521 IMEPIC samples (GSE152026) normalized with dasen wateRmelon method, 656 IM450K samples (GSE40279) and 4 additional IMEPICv2 control samples (GSE222919) normalized with Illumina method. The specificity reached 100% for all the classifiers, apart from Kabuki^SVM_Funnorm^ and Kabuki^SVM_Noob^ which predicted some false positives when tested on the GSE152026 dataset*.* In both **A** and **B,** the probability represents the prediction probability of being classified as a Sotos or Kabuki sample by the corresponding classifiers

Finally, to further assess the specificity of the DMR-esig, the best predictive models for Sotos and Kabuki syndrome were tested on three separate cohorts of control samples. We used 3 separate control cohorts obtained from IM450K, IMEPIC and IMEPICv2, to assess any influence from the technology. The Sotos^PLR^ achieved a specificity of 100% in both IM450K, IMEPIC and IMEPICv2 samples for all three best normalization methods. Instead, the best Kabuki predictive model (Kabuki^SVM^) achieved 100% of specificity only when using the model trained on data normalized with Illumina normalization method (Fig. 4). However, it is important to highlight that the other two normalization methods achieved 100% of specificity when considering only the 450 K and EPICv2 cohorts. These two cohorts of samples were indeed normalized using Illumina normalization as described in “Materials and methods”, while the cohort of EPICv1 samples was normalized using the dasen wateRmelon. This different preprocessing could explain why the EPIC prediction probability distribution is more spread and shifted toward 1. This evidence is confirmed by the previous section (Fig. 2) where, looking at the heatmap for Kabuki syndrome, it was clear that the performance of the best predictive model (SVM) is highly dependent on the normalization method adopted. Instead, the best Sotos predictive model was PLR, which is much more stable and robust to the normalization method adopted. That could partially explain the presence of false positives predicted by the best Kabuki predictive model. In addition, we applied our models to a cohort of unresolved cases previously described (see Methods). As these samples were normalized using the BMIQ method, the same considerations described above apply and some false positives can be expected. Nevertheless, two samples (GSM2366406, GSM2366710) were consistently predicted as positives by Kabuki^SVM^ with a prediction probability of 0.78, 0.92, 0.88 and 0.86, 0.80, 0.82 for Funnorm, Illumina, and Noob methods, respectively. On the other hand, one sample (GSM2366674) was consistently predicted as positive by the best Sotos predictive model with a prediction probability of 0.87, 0.96, and 0.87 when using Funnorm, Illumina, and Noob functions, respectively (Fig. 4A). Interestingly, those 3 patients showed a phenotype that was coherent with the predictions of our models (see Supplementary Data 1 in Barbosa et al. (2018)). Both GSM2366406 and GSM2366710 presented facial dysmorphism and congenital heart defects. In addition, GSM2366406 showed moderate intellectual disability that was not described for GSM2366710. The predicted Sotos sample (GSM2366674) was a newborn (age = 0) of short stature at the time of the study and suffered from hydrocephalus, heterotaxy and inguinal hernia.

### Functional analysis of DMRs-episignature and interpretability of the models

**Fig. 5:**
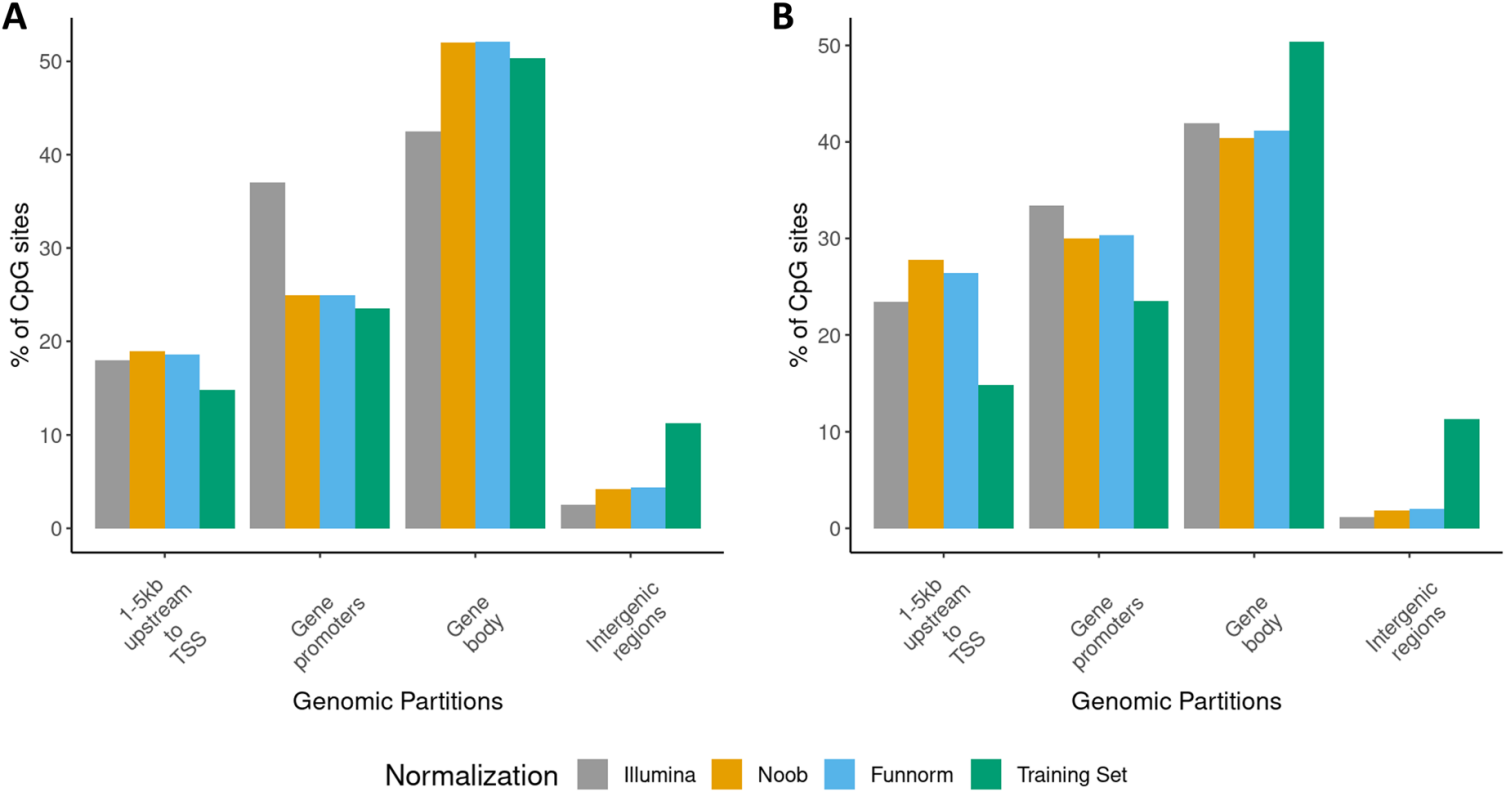
**A** and **B**, respectively, represent the genomic partitions of the CpGs included in the Sotos^DMRs^ (**A**) and Kabuki^DMRs^ (**B**). The CpGs included in the DMRs were also compared to the genomic partitions of all the CpGs included in the Sotos (**A**) and Kabuki (**B**) training sets (see Table 2)

A functional analysis was performed to elucidate the pathophysiology of Kabuki and Sotos Syndromes. The genomic locations of the DMR-esigs for Illumina, Noob, and Funnorm normalization methods show that the DMRs are located mostly in the gene bodies and promoters. Furthermore, the genomic partition analysis shows an enrichment of CpGs overlapping gene promoters and regions 1–5 kb upstream the TSS compared to the training sets used for both the Kabuki and Sotos datasets (Fig. 5 green bars). This result is consistent with previous published studies (Choufani et al. 2015; Butcher et al. 2017).

DAVID (Huang et al. 2009) was used to identify the biological processes most enriched with respect to the Kabuki and Sotos DMR-esig. We looked at the common genes between the three normalization methods for each syndrome and used them for the enrichment analysis. For the Kabuki dataset, the results showed an enrichment in genes implicated in the embryonic organ and skeletal development and morphogenesis (Additional File 3). Concerning Sotos dataset, the results highlighted an enrichment in nervous system development, system development, and developmental process, as well as cell–cell adhesion (Additional File 4), which is coherent with Sotos syndrome. These results confirmed the results already obtained in previous studies using DMC-esig instead of DMR-esig (Choufani et al. 2015; Butcher et al. 2017). Furthermore, the list of genes used for this analysis is shown in Additional File 5. Some of the HOXA cluster genes were highly relevant in the enrichment analysis of Kabuki syndrome and generally highly present in the genes overlapping the DMRs. This finding confirms previous studies (Butcher et al. 2017). On the other hand, some genes of the PCDHGA subfamily were found to overlap Sotos^DMR^ episignatures. This subfamily of genes encodes for large transmembrane proteins that are critical for processes such as cell-signaling (Peek et al. 2017). Furthermore, in a previous study, the researchers hypothesized that misregulation of this subfamily of proteins can contribute to the neurodevelopmental phenotype of Sotos syndrome (Bondhus et al. 2022). Taken together, these findings confirm the importance of the interpretation of episignatures and underline the capability of DMR-esigs to provide the same insights as DMC-esigs, with a higher and broader view of the mechanisms that are involved in the etiology of a disease.

Finally, the coefficients of Sotos^PLR_Noob^, Sotos^PLR_Funnorm^ and Sotos^PLR_Illumina^ were obtained in order to assess the importance of the DMR predictors on the model classification. This was possible for the Sotos syndrome since PLR is an interpretable model. Concerning Kabuki syndrome, the SVM with a polynomial kernel does not return interpretable coefficients. In the Additional File 6 the features importance is reported for each of the three best normalization methods. In both Sotos^PLR_Funnorm^ and Sotos^PLR_Noob^, the two most important DMR predictors considering the coefficient absolute value were the same and matched AIRE and ZNF833P genes. Concerning Sotos^PLR_Illumina^, the two most important DMRs, yet considering the absolute value of the coefficients, were the DMR_9 and the DMR_104 which match, respectively, AIRE and TOPAZ1 genes. Regarding these genes, no important evidence that links them to Sotos syndrome has been found in the literature.

## Discussion

Episignature are powerful tools to enable patient diagnosis and classification of variants of unknown significance (VUS). However, due to their relative novelty, no framework on how to build them has been described. Nevertheless, efforts from one group lead to the first guidelines on how to properly design cohorts for episignature discovery (Chater-Diehl et al. 2021). In this work, we built on this and previous work and propose a thorough analysis of how the normalization method, classification model, and missing data may affect episignature performances. We chose to evaluate the methods available in the *minfi* package, as it is the most popular tool to preprocess DNAm data from Infinium arrays. Indeed, several normalization methods are implemented in *minfi****,*** including the one from the equally popular Illumina GenomeStudio software. We used two episignatures for Kabuki and Sotos syndromes implemented in Epigencentral (Turinsky et al. 2020) as the training datasets were publicly available. The datasets consist of peripheral blood samples, which have exhibited a strong correlation in terms of DNAm profiling with brain regions in individuals affected by NDDs (Gregory et al. 2009; Murphy 2005). In fact, since the underlying events leading to most NDDs occur early in embryonic development, DNAm patterns associated with these syndromes have been detected in various tissues of affected individuals, including peripheral blood (Aref-Eshghi eyt al. 2020). In addition, given the practical constraints of obtaining brain tissue from living individuals, peripheral blood serves as a valuable, accessible, and minimally invasive alternative for diagnostic purposes (Aref-Eshghi 2020). However, peripheral blood samples are also characterized by a high presence of noise that can bias the results. For this reason, the entire preprocessing procedure of the samples holds an important task and must be performed carefully in order to reduce such noise. In addition, we designed a new strategy for episignature discovery based on DMRs identification. This strategy was motivated by the observation that episignatures are usually based on methylation profiles assessed by microarray technologies. Methylation arrays are subject to poor probe hybridization and new version releases that drop or add new CpG loci, which may lead to missing values. In classification tasks, the issue of missing data is usually resolved by imputation. However, this may cause noise or error in the models. This is particularly the case in a biological context, where imputation should only be performed under a strong hypothesis. We observed that with the new iterations of the Infinium arrays (IM450K; IMEPIC; IMEPICv2) there were already missing CpGs in both Kabuki and Sotos published episignatures (i.e., from IM450K to IMEPICv2: 87% concordance for Kabuki, 91% for Sotos). The new paradigm of DMR-based episignatures that we propose (see Methods) tackles this issue by considering the median methylation of the CpGs within a region, therefore decreasing the reliance on individual loci. Unsupervised hierarchical clustering is a classical way to assess the capacity of a signature to differentiate between cases and controls. Using this method, we saw that both types of episignatures (both DMR-esig and DMP-esig), for all the six normalization methods, were able to correctly cluster the Sotos and the Kabuki training sets. In addition, we showed that the DMR-esig retained the ability to further refine the clustering of patients showing a different profile within a syndrome. We then explored the performances of the three most commonly used classification models. SVMs are usually the model of choice for episignatures classification tasks (Choufani et al. 2015; Butcher et al. 2017; Aref-Eshghi et al. 2020; Turinsky et al. 2020; Verberne et al. 2022; Coenen-van der Spek, et al. 2023). However, there is no existing work on how other models performed in comparison. Therefore, we chose to implement SVM, RF, and PLR models and evaluated their performances using the MCC. We showed that in both DMC-esig, PLR was the only classifier reaching an MCC of 1 in every setting, whereas SVM or RF classifiers’ performances depended on the method used to normalize data. Using DMR-esig, PLR showed the overall best performance on the Sotos testing cohort, while SVM showed the best performance on the Kabuki testing cohort. These results may be due to the fact that those two syndromes differ in the size effect in methylation levels between patients and control. PLR may perform better to discriminate between large effects where SVM performs better with less strong differential methylation. We also highlighted that model performances on training sets normalized with the Raw, Swan, or Quantile methods were almost always worse than Illumina, Noob, or Funnorm. Therefore, we would advise against the use of those methods during data preprocessing. We then analyzed the influence of missing data on the model performances. As discussed above, missing data may represent an increasing challenge when episignatures are developed on deprecated technologies. We assessed the performances of DMC-esig and DMR-esig in the testing sets showing increased missing data (5, 10, 20 and 30% of missing probes). Our results indicated that both DMC-esig and DMR-esig are valid options in a classification task in the context of rare disorders affecting DNAm. However, with an increasing percentage of missing probes in the testing set, the models based on DMR-esig slightly outperformed the ones based on DMC-esig in terms of mean MCC. These results highlight that DMR-based episignatures are more robust to missing data. Therefore, we suggest the readers to envision this approach when building episignatures. In addition, DMR-based episignatures are less likely to become deprecated as new technologies to measure DNA methylation become available (e.g., reduced representation bisulfite sequencing (RRBS), enzymatic methyl-seq (EM-seq), Oxford Nanopore Technologies). Nevertheless, we want to draw attention to the fact that other methods for DMR detection exist (Mallik 2019; Zheng et al. 2022). Those methods have been shown to outperform bumphunter and could be valid alternatives in the implementation of DMR-esigs. In the two syndromes presented here, we saw that using DMRcate (Peters et al. 2015), the best-performing software in (Mallik 2019), to identify DMRs did not lead to significant change in performance. It slightly increased the MCC on the testing sets when using Illumina, Noob and Funnorm normalization methods (Additional Fig. 6), slightly decreased when using Raw, Swan and Quantile normalization methods (Additional Fig. 6) and slightly decreased the specificity on the GSE40279 control cohort (0.98, 0.98 and 0.99 when using Kabuki^SVM_Illumina^, Kabuki^SVM_Noob^ and Kabuki^SVM_Funnorm^, respectively, while it remains equal to 1 when using Sotos^PLR_Illumina^, Sotos^PLR_Noob^ and Sotos^PLR_Funnorm^ classifiers). This could be due to the fact that the effect size in those two syndromes were close to the threshold at which bumphunter performs best (Mallik 2019; Zheng et al. 2022). In addition, we saw that bumphunter-based DMR-esig classifiers performed well. However, we would advise comparing the performances of those different methods when implementing a DMR-esig in other disorders.

## Conclusions

This work highlights three key points that are important to consider when building a new episignature. First, the choice of the best model is highly dependent on the dataset, and particularly on the type of disease and features. For this reason, it is better to provide a unique model that, during the building procedure, was shown to be the most accurate and robust to missing data and normalization methods. In addition, in the context of clinical diagnosis, it is important to consider the interpretability of the model, and it is always better to choose an interpretable model (e.g., linear SVM, PLR) when possible. Indeed, being able to access features importance allows to better characterize the underlying biological mechanisms of the episignature. Second, the choice of normalization method is necessary and must be done carefully when training a model. Overall, from our analysis, it is preferable to use Funnorm, Noob or Illumina as normalization functions and avoid the use of Raw, Quantile, and Swan. The first three have been shown to maximize the accuracy independently of the type of model or disease. Finally, the use of DMRs instead of DMCs as features to train a classification model has been demonstrated to be a valid, accurate, and robust alternative to currently adopted episignatures. This new approach does not need any imputation algorithm to deal with missing data, nor, when a new Illumina array version is released, to repeat the procedure of building a new model that is trained on common CpGs between the old and the new array version.

## Materials and methods

### Subjects and cohort

The study includes peripheral blood from 26 samples collected at the ULB Erasme Hospital Center for Human Genetics (Brussels, Belgium). Informed consent was obtained from all research participants according to the protocol approved by the Ethics committee of the Erasme Hospital. These are composed of 22 controls, 1 patient with a confirmed diagnosis of Kabuki syndrome (OMIM: #147920) and 3 patients with a confirmed diagnosis of Sotos syndrome (OMIM: #117550) through the identification of class IV or V variants by genetic testing. Control specimens were healthy individuals without any developmental delay, intellectual disability, or congenital anomalies. Additional peripheral blood datasets were retrieved from the gene expression omnibus. They include data used to generate the Kabuki (IM450K, GSE97362) and Sotos (IM450K, GSE74432) episignatures (Choufani et al. 2015; Butcher et al. 2017), a cohort of 489 unresolved cases (IM450K, GSE89353) (Barbosa et al. 2018), 521 control samples (IMEPIC, GSE152026) (Noguera-Castells et al. 2023), 656 control samples (IM450K, GSE40279) and 4 additional control samples (IMEPICv2, GSE222919) (Teschendorff et al. 2013).

### DNA methylation experiment

Whole-blood DNA samples were bisulfite converted using the EZ DNA Methylation™ Kit from Zymo Research according to manufacturer standard procedure. The sodium bisulfite converted DNA was then hybridized to the Illumina Infinium MethylationEPIC v1.0 BeadChip to interrogate over 850,000 CpG sites in the human genome. The scan of the array has been done by the BRIGHTcore facility of UZ Brussels.

### Training and testing sets

Training and testing sets for the Kabuki and Sotos episignatures were derived from datasets GSE97362 and GSE74432, respectively. Additional Files 7 and 8 contain the GEO ID of the training sets for the Kabuki and Sotos cohort, respectively. The training sets were balanced with the same number of control and case samples (Table 2). Two additional testing sets composed of IMEPIC samples generated at the ULB Erasme Hospital Center for Human Genetics (Brussels, Belgium) were used to assess the model performances on external IMEPIC data (Table 2).

**Table 2:** Description of the three datasets used in this work. Two testing sets based on 450 K and EPIC samples have been used to test the performances of the classifiers. One training set based on 450 K samples has been used to train the models

Array	Source	Scope	*N*	Syndrome	#Cases	#Controls
450 K	GSE74432	Training	38	Sotos	19	19
450 K	GSE74432	Testing	78	Sotos	19	59
EPIC	Erasme Hospital	Testing	25	Sotos	3	22
450 K	GSE97362	Training	22	Kabuki	11	11
450 K	GSE97362	Testing	93	Kabuki	8	85
EPIC	Erasme Hospital	Testing	23	Kabuki	1	22

The Sotos case training set is composed of 8 females and 11 males, with an average age of 11.67 ± 10.97 years (range 0–41 years), while the control group is composed of 12 females and 7 males with an average age at sample collection of 10.94 ± 5.45 years (range 3–18 years) (Additional File 8). The Kabuki case training group is composed of two females and nine males with an average age at sample collection of 13.64 ± 5.64 years (range 1–20 years), while the control group is composed of two females and nine males with an average age at sample collection of 10 ± 3.43 years (range 5.5–13 years) (Additional File 7). The testing cohort includes the rest of the respective datasets without the samples with variants of unknown significance (VUS), as well as the data from Erasme Hospital.

### Data preprocessing

Data from the original articles and in-house data were processed using the *minfi* library in *R* (Aryee et al. 2014). Probes with low-quality signals (detection *p* value > 0.01), cross-reactive probes, probes harboring single nucleotide polymorphisms (SNPs), and probes on sex chromosomes were removed. The cross-reactive probes were removed using the R library *maxprobes.* The total number of probes retained for each dataset is shown in Table 3. Normalization of the data was performed using the 6 different methods available in *minfi* using default parameters: Noob (Triche et al. 2013), Illumina, Funnorm (Fortin et al. 2014), Raw (no normalization), Quantile (Touleimat and Tost 2012) and Swan (Maksimovic et al. 2012). The Illumina normalization method implemented in *minfi* is the same method used in Choufani et al. (2015) and Butcher et al. (2017) to normalize the beta-values. The normalization, as for the preprocessing, was performed separately on training and testing sets in order to reduce the bias and avoid overestimating the accuracy of the classifiers in the testing sets. Quality Control (QC) of the samples was assessed using *minfi* package (Aryee et al. 2014): no bad quality samples were found, thus, all samples were kept for the analysis. We used the Illumina annotations based on the *hg19 genome version* in order to have the same, comparable positions and annotations for all array versions.

**Table 3:** Nnumber of probes remaining after preprocessing and filtering together with their percentages

Dataset	Before preprocessing	After preprocessing	% of probes removed
Sotos 450 K-Training	485,512	420,335	13.4%
Sotos 450 K-Testing	485,512	419,899	13.5%
Sotos EPIC-Testing	865,859	773,785	10.6%
Kabuki 450 K-Training	485,512	420,529	13.4%
Kabuki 450 K-Testing	485,512	432,492	11%
Kabuki EPIC-Testing	865,859	773,906	10.6%

### Differentially methylated regions (DMRs) episignatures

To identify DMRs in the Kabuki and Sotos training sets, we used the *ChAMP library* available in R on the training set (Tian et al. 2017). Particularly, the *DMR* function has been adopted specifying *bumphunter* as DMRs detection function (Jaffe et al. 2012). The following non-default parameters were used: *number of bootstraps* = *1000*; *minimum number of probes per DMR: 10*. For both syndromes, this function was run on the training set 6 times: once per normalization method. The *cutoff parameter* was decided by the software, except for the Sotos dataset, where we applied a more stringent filter in order to reduce the number of DMRs found. Thus, the *cutoff parameter* was set as 0.7 for Sotos syndrome and kept as *0.292* for Kabuki syndrome. Only DMRs with an adjusted *p* value < 0.05 were retained. Once the DMRs were computed, the median methylation value of the probes included in each DMR was extracted and considered as the methylation value for that DMR. The methylation values of the DMRs were computed separately for the training and the testing sets.

### Hierarchical clustering and UMAP

Hierarchical clustering was performed using the *seaborn clustermap* function, with *average* as linkage method and *cityblock (manhattan)* as the distance metric to cluster samples, which is preferred for Euclidean distance for high-dimensional application (Aggarwal et al. 2001). Python *umap* package was used to visualize the samples in a two-dimensional space, using beta-values from the DMC-esig or median beta-values from the DMRs in the DMR-esig, respectively. Data were scaled with *Python scikit-learn StandardScaler function* prior to using UMAP.

### Machine learning classifiers for discriminating between syndrome and non-syndrome samples

**Fig. 6:**
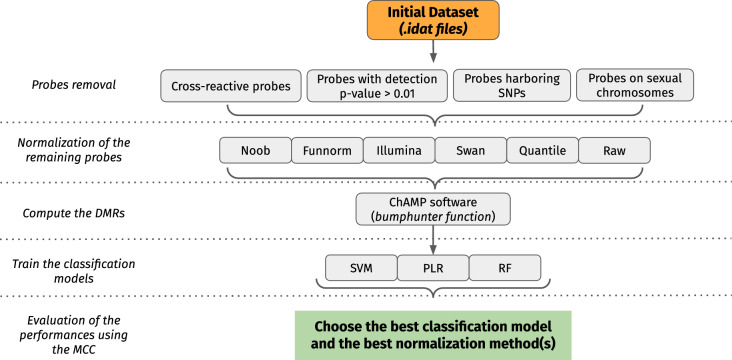
Procedure adopted to build classification models based on DMR-esigs for both Kabuki and Sotos syndromes

Support Vector Machine (SVM), Random Forest (RF), and Logistic Regression regularized with penalization term (PLR) were trained either on the beta-values of the DMC-esig, or on the median beta-values of the DMRs constituting the DMR-esig. Moreover, for each syndrome, the models were trained on each of the six normalized training sets as described above. The optimization algorithm of PLR was set as *liblinear* within the *solver* parameter, while we kept the default *L2 penalty* with a *dual* formulation. The hyperparameters were specified within a GridSearch Analysis and the optimal ones were chosen through a leave-one-out cross-validation (LOOCV) (Additional File 9). The classifiers were then tested on the test sets to assess their performances on new, unknown samples. The prediction probabilities were also extracted from the results. Matthew’s correlation coefficient (MCC) was used to evaluate the models’ performances. Indeed, MCC is preferred to the F1 score in binary classification tasks due to its higher robustness to imbalanced datasets (Chicco and Jurman 2020). Model training, testing, and performance assessment were done using *scikit-learn.* The procedure adopted to build classification models for both Sotos and Kabuki syndrome is summarized in Fig. 6.

### Testing robustness for missing probes

To avoid missing values in the DMC-esig, *nan* resulting from CpGs removal (due to new array version and/or low-quality signal probes) were imputed using *pandas.fillna(method* = ‘*ffill’)*. In order to test the robustness of the different classifiers, we also removed from all the testing datasets 5, 10, 20 or 30% of CpGs from the whole array after normalization. We then imputed the missing probes with the same procedure adopted above. This process simulates a scenario in which the release of new technology and the removal of low-quality signal probes introduce missing values in the samples to be classified with existing models. In the DMR-esig, no imputation was performed and for each DMR the median methylation value has been computed on the remaining probes within that DMR. Then, prediction performances and probabilities for each model have been extracted in order to evaluate the robustness of both approaches. We repeated this process ten times, using different random seeds to select the CpGs to remove and computed the mean and the standard deviation of the MCC from all individual performance evaluations.

### Validation of the DMR-esig models on external datasets

To assess the specificity of the models trained on DMR-esig across all array versions, a cohort of 521 IMEPIC control samples (GSE152026), 656 IM450K control samples (GSE40279) and 4 additional IMEPICv2 control samples (GSE222919) were used. Furthermore, the models were applied to a cohort of 489 IM450K unresolved cases (GSE89353). Data from GSE89353, GSE222919, GSE152026, and GSE40279 were retrieved already preprocessed and normalized using BMIQ (Quantile), Illumina GenomeStudio, the wateRmelon *dasen* function and Illumina GenomeStudio, respectively, as described in the original studies (Barbosa et al. 2018; Noguera-Castells et al. 2023; Teschendorff et al. 2013; Hannum et al. 2013). Beta-values were used as such without any further processing.

### Functional analysis

All analyses were performed using *R (version 4.1.2).* For both Kabuki and Sotos DMR-esigs using Illumina, Noob and Funnorm normalization methods, we extracted the CpGs belonging to the DMRs of each episignature. Then, the CpGs were annotated to the genome assembly GRCh37 (*hg19*) using *annotatr (v. 1.24.0)*. Genic annotations included regions 1-5 Kb upstream of the transcription start site (TSS), gene promoters (< 1 Kb upstream of the TSS), intergenic regions, 5’UTRs and 3’UTRs, exons, introns and intron–exon boundaries, using the following built-in annotations: ‘hg19_basicgenes’, ‘hg19_genes_intergenic’ and ‘hg19_genes_intronexonboundaries’. The UTRs, exons, introns and intron–exon boundaries were considered part of the “gene body” category. The CpGs genomic annotations of the episignatures were then compared to the CpGs genomic annotations of the entire corresponding training set in order to understand which regions are most enriched in the DMR/esigs.

Gene ontology enrichment analysis was performed using *DAVID (*Huang et al. 2009) to identify the biological processes most enriched. The genes used for the enrichment analysis were only the in-common genes among the DMR^Illumina^, DMR^Noob^ and DMR^Funnorm^ episignatures. Enriched terms were filtered at adjusted *P* value < 0.05 (Benjamini–Hochberg procedure).

### Software versions

Analyses were performed either in Python (version: 3.8.10) or R (version 4.1.2).

The following version of the packages were used: *minfi (version 1.40.0); maxprobes (version 0.0.2); ChAMP (version 2.24.0); seaborn (version 0.12.2); umap (version 0.5.3); scikit-learn (version: 1.2.2); pandas (version: 1.5.3).*

### Supplementary Information

Below is the link to the electronic supplementary material.

Supplementary file1 (ZIP 4431 KB)

## Data Availability

Public datasets used in the study are described in the Methods section. Data generated in Erasme Hospital can be obtained from the corresponding author upon a reasonable request.

## Abbreviations

DNAm: DNA methylation
DMC: Differentially methylated cytosine
DMC-esig: DMC-based episignature
DMR: Differentially methylated region
DMR-esig: DMR-based episignature
EWAS: Epigenome-wide association study
IM450K: Infinium HumanMethylation 450 K BeadChip
IMEPIC: Infinium HumanMethylation EPIC BeadChip
IMEPICv2: Infinium HumanMethylation EPICv2 BeadChip
LOOCV: Leave-one-out cross-validation
MCC: Matthew’s correlation coefficient
NDD: Neurodevelopmental disorder
PLR: Penalized logistic regression
QC: Quality control
RF: Random forest
SNP: Single nucleotide polymorphisms
SVM: Support vector machine
VUS: Variant of unknown significance
