# Supplementary material for: Comprehensive evaluation of the implementation of episignatures for diagnosis of neurodevelopmental disorders (NDDs)
Source: Hum Genet. 2023 Oct 27;142(12):1721–35. doi: 10.1007/s00439-023-02609-2 (PMC10676303; doi:10.1007/s00439-023-02609-2)
Supplement: Supplementary file 1 — Supplementary file1 (ZIP 4431 KB) [file 439_2023_2609_MOESM1_ESM.zip › Additional Files/Additional_Figures.pdf]

Additional Figure 1

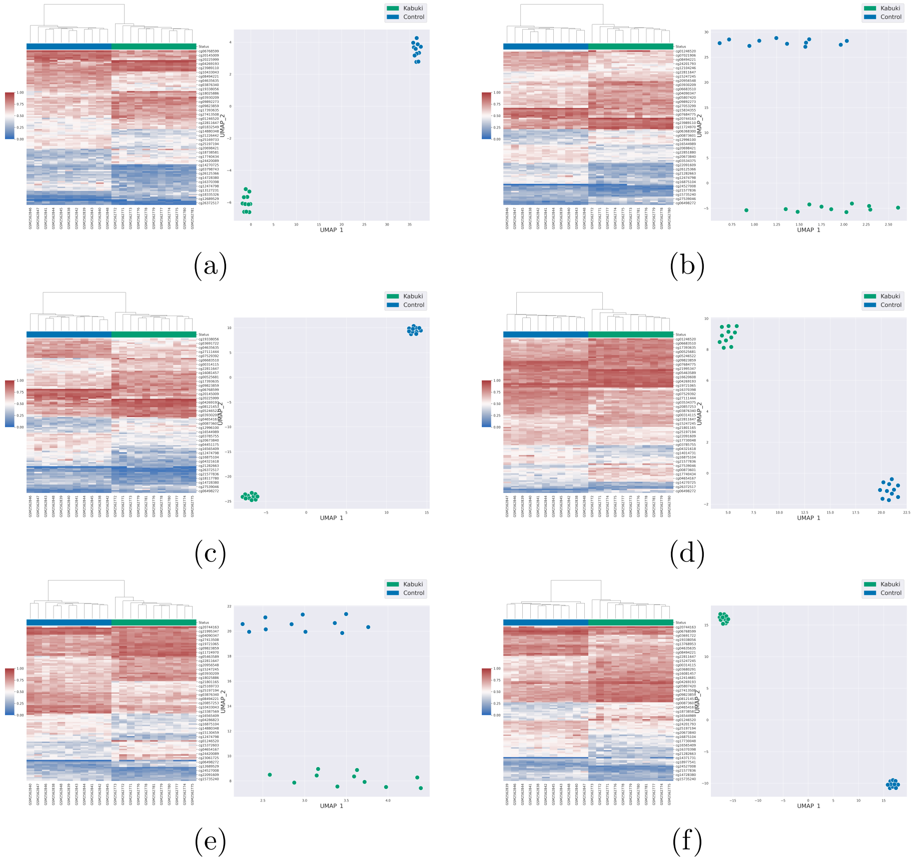

Additional Figure 1: On the right the UMAPs of the samples according to the DMC-esigs normalized with the six normalization methods (Funnorm (a), Illumina (b), Noob (c), Quantile (d), Raw (e) and Swan (f)). On the left the corresponding heatmaps and the dendrograms resulting from the hierarchical clustering. In green the Kabuki case samples, in blue the control samples.

Additional Figure 2

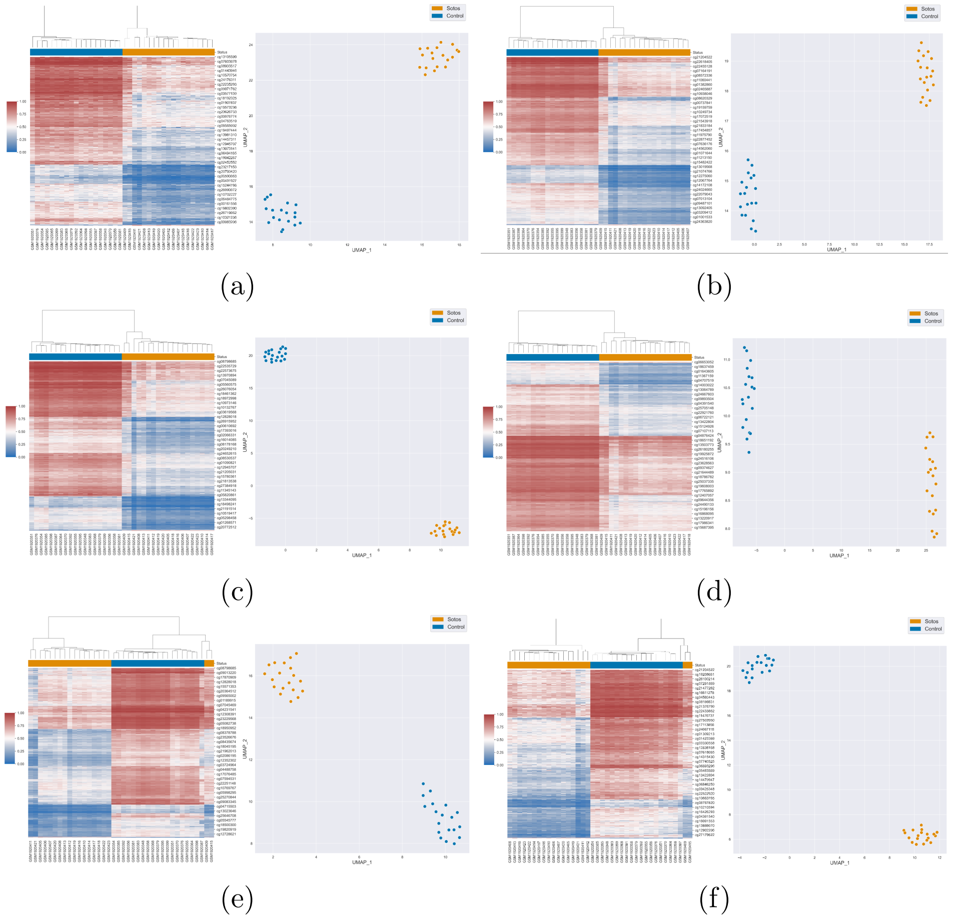

Additional Figure 2: On the right the UMAPs of the samples according to the DMC-esigs normalized with the six normalization methods (Funnorm (a), Illumina (b), Noob (c), Quantile (d), Raw (e) and Swan (f)). On the left the corresponding heatmaps and the dendrograms resulting from the hierarchical clustering. In orange the Sotos case samples, in blue the control samples.

Additional Figure 3

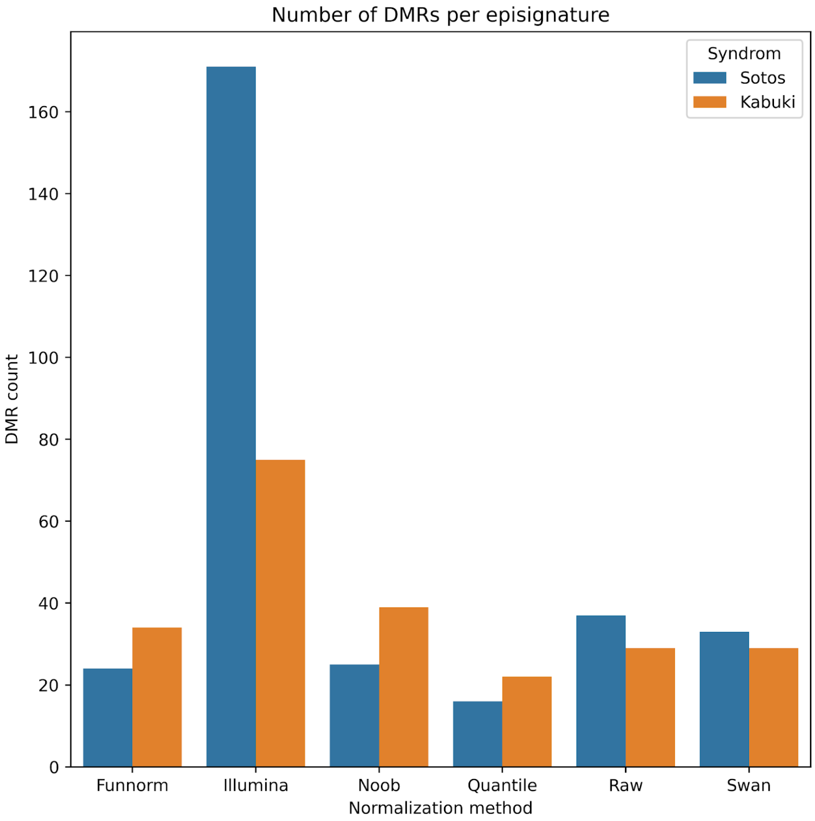

Additional Figure 3: Number of DMRs per normalization method for both Sotos and Kabuki syndromes.

Additional Figure 4

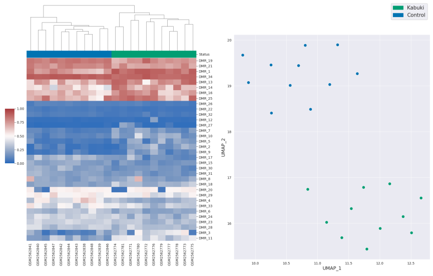

(a)

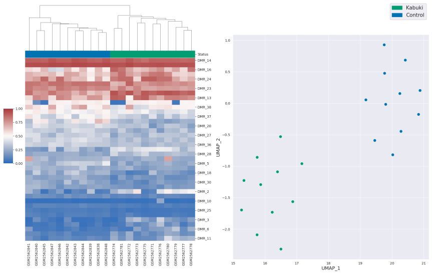

(b)

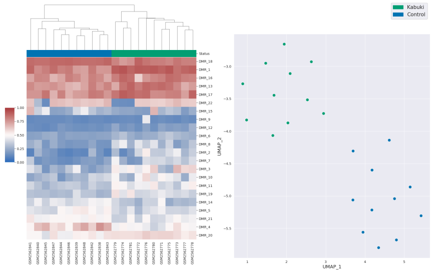

(c)

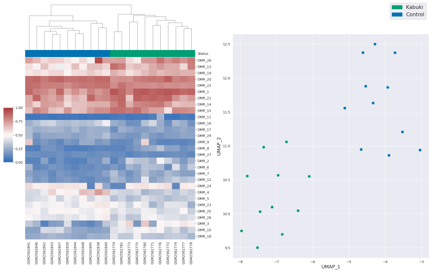

(d)

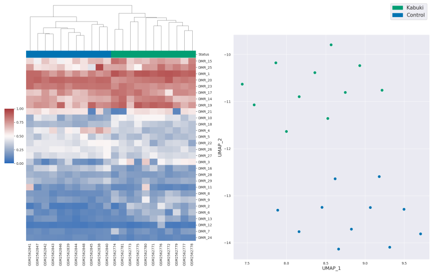

(e)

Additional Figure 4: On the right the UMAPs of the samples according to the DMR-esigs normalized with the six normalization methods apart from Illumina function which was included in the main text (Funnorm (a), Noob (b), Quantile (c), Raw (d) and Swan (e)). On the left the corresponding heatmaps and the dendrograms resulting from the hierarchical clustering. In green the Kabuki case samples, in blue the control samples.

Additional Figure 5

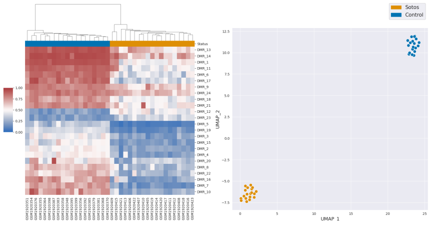

(a)

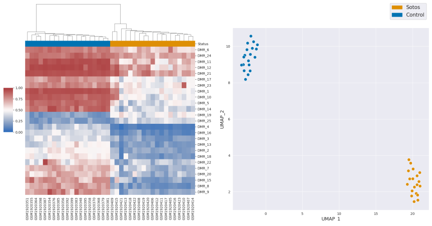

(b)

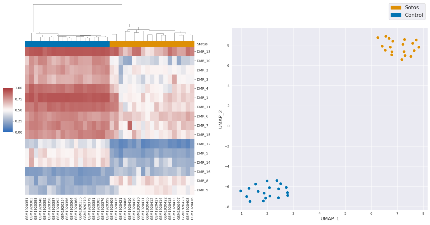

(c)

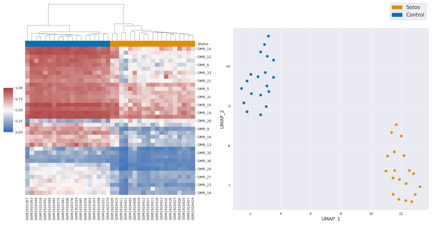

(d)

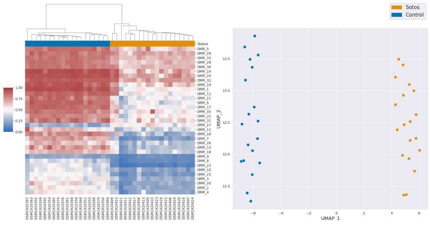

(e)

Additional Figure 5: On the right the UMAPs of the samples according to the DMR-esigs normalized with the six normalization methods apart from Illumina function which was included in the main text (Funnorm (a), Noob (b), Quantile (c), Raw (d) and Swan (e)). On the left the corresponding heatmaps and the dendrograms resulting from the hierarchical clustering. In orange the Sotos case samples, in blue the control samples.

Additional Figure 6

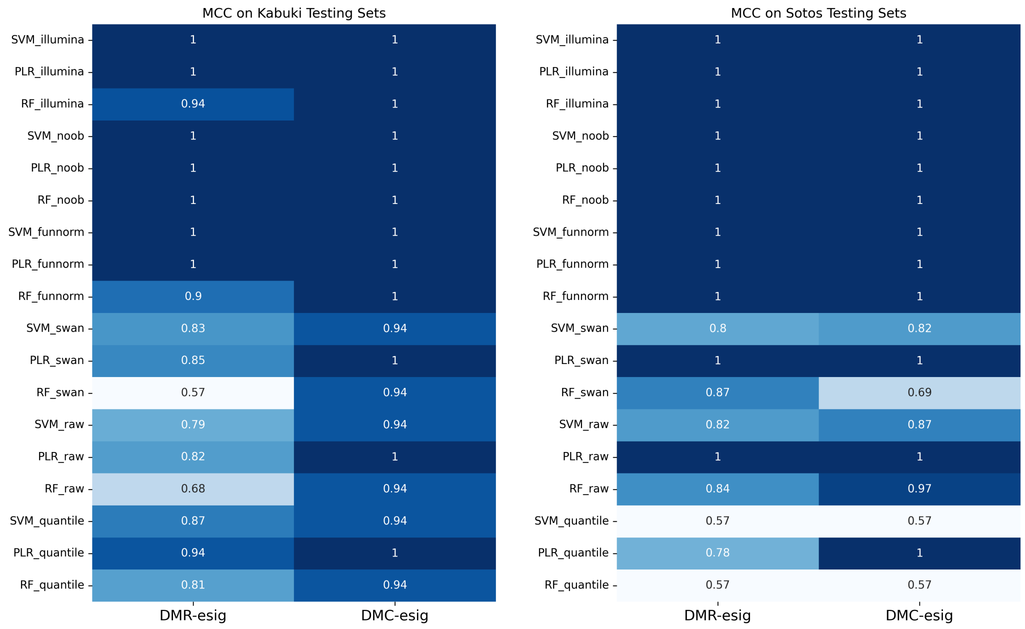

Additional Figure 6: The heatmaps represent the MCC values on Kabuki (left) and Sotos (right) Testing Sets composed of both public IM450K samples and IMEPIC samples generated at Erasme Hospital. In the heatmaps, the values represent the MCC computed after the predictions given by the three models tested in this study (SVM, PLR, RF) when trained on DMR-esig and DMC-esig generated on training sets previously normalized with the six different normalization methods available in minfi library. Here, the DMRs were identified using the DMRcate software.
